# Patient perceptions regarding benefits of single visit scale and polish: a randomised controlled trial

**DOI:** 10.1186/1472-6831-13-50

**Published:** 2013-10-03

**Authors:** Clare Jones, Tatiana V Macfarlane, Keith M Milsom, Philip Ratcliffe, Annette Wyllie, Martin Tickle

**Affiliations:** 1 School of Dentistry, The University of Manchester, Coupland 3 Building, Oxford Road, Manchester M13 9PL, UK; 2 Division of Applied Medicine, School of Medicine and Dentistry, University of Aberdeen, Polwarth Building, Foresterhill, Aberdeen, AB25 2ZD UK; 3 Cheshire & Merseyside Centre, Public Health England, Chester, UK; 4 Woodlands Dental Practice, 493 Old Chester Rd, Dacre Hill, Birkenhead CH42 4NG, UK; 5 Martins Lane Dental Practice, 1-3 Martins Lane, Wallasey WIRRAL CH44 1BA, UK

**Keywords:** Practice-based RCT, Routine scale and polish, Patient-reported outcomes

## Abstract

**Background:**

Single visit scale and polish is frequently carried out in dental practices however there is little evidence to support (or refute) its clinical effectiveness. The purpose of this research was to compare patient-reported outcomes between groups receiving a scale and polish at 6-, 12-, and 24-month intervals. Outcomes recorded included participants’ subjective assessment of their oral cleanliness; the perceived importance of scale and polish for oral health and aesthetics; and frequency at which this treatment is required.

**Methods:**

A practice-based randomised control trial was undertaken, with a 24-month follow-up period. Participants were healthy adults with no significant periodontal disease (BPE codes <3) randomly allocated to three groups to receive scale and polish at 6-, 12-, or 24-month intervals. Patient-reported outcomes were recorded at baseline and follow-up. Oral cleanliness was reported using a 5-point scale and recorded by examiners blinded to trial group allocation. A self-completed questionnaire enabled participants to report perceived importance of scale and polish (5-point scale), and required frequency of treatment (6-point scale). The main hypothesis was that participants receiving 6-monthly scale and polish would report higher levels of oral cleanliness compared to participants receiving scale and polish at 12- and 24-month intervals.

**Results:**

369 participants were randomised: 125 to the 6-month group; 122 to the 12-month group; and 122 to the 24-month group. Complete data set analysis was carried out to include 107 (6-month group), 100 (12-month group) and 100 (24-month group) participants. Multiple imputation analyses were conducted where follow-up data was missing. The difference in the proportions of participants reporting a 'high’ level of oral cleanliness at follow-up was significant (Chi-squared P = 0.003): 52.3% (6-month group), 47.0% (12-month group) and 30.0% (24-month group). Scale and polish was thought to be important by the majority in each group for keeping mouths clean and gums healthy, whitening teeth, and preventing bad breath and tooth decay; there were no statistically significant differences between groups at follow-up. Most participants at follow-up thought that the frequency of scale and polish should be “every 6 months” or more frequently: 77.9% (6-month group), 64.6% (12-month group), 71.7% (24-month group); differences between groups were not statistically significant (Chi squared P = 0.126). The results suggest that participants in the 24-month trial group were more likely to choose a scale and polish interval of “once a year” or less frequently (OR 2.89; 95% CI 1.36, 6.13).

**Conclusions:**

The majority of healthy adults regarded 6-monthly single-visit scale and polish as being beneficial for their oral health. Receiving the treatment at different frequencies did not alter this belief; and those with the longest interval between scale and polish provision perceived that their mouth was less clean. In the absence of a strong evidence base to support (or refute) the effectiveness of single-visit scale and polish, the beliefs and preferences of patients regarding scale and polish may be influential drivers for maintaining provision of this treatment.

## Background

Patients attending primary care dental practices may receive 'scale and polish’ ('oral prophylaxis’) as part of their dental care; the treatment is traditionally linked to a routine, usually 6-monthly, dental check-up [1]. This is a commonly-provided, and therefore costly, procedure. Currently, a little under half of the courses of National Health Service (NHS) dental treatment delivered by primary care practitioners in England include provision of a scale and polish [2]. In Scotland, where a fee-for-item remuneration system exists, simple periodontal treatment (93.6% of which is single-visit scale and polish) accounts for 45 in every 100 courses of treatment [3]. Dental practitioners are advised to give accompanying oral health advice to promote effective patient self-care [4]; it has been suggested that the professional clinical intervention has limited value if adjunctive advice is not provided [5].

A recent Cochrane systematic review investigated 'routine scale and polish for adult periodontal health’ and was unable to identify strong evidence to support (or refute) beneficial (or harmful) effects of the treatment; furthermore, the authors were unable to conclude the optimal frequency at which scale and polish should be provided [6]. Given the lack of evidence regarding clinical benefits of routine scale and polish provision on (normatively-measured) periodontal health [6-8], it is likely that treatment provision is largely based upon clinicians’ professional knowledge and experience, and the personal views of patients; both groups cite non-clinical benefits such as improved aesthetics as rationales for treatment [9]. The importance of scale and polish on periodontal health from the perspective of the patient has not previously been robustly assessed and the recommendations of the Cochrane review team included a requirement for further primary care-based research to investigate patient-reported outcomes in addition to objective clinical measurements [6].

A practice-based randomised controlled trial (RCT) was carried out in the North West of England (during the period February 2006 - September 2009) to investigate the health outcomes of single-visit scale and polish when this is provided at different frequencies. The objective clinical findings (gingival bleeding, plaque and calculus presence) have been published separately [8]; this paper presents the patient-reported outcomes of the trial and views of participants regarding the importance of receiving this commonly-provided, yet ill-defined treatment. The objectives of the study were to compare subjectively-assessed oral cleanliness, subjectively-perceived importance of scale and polish and its effects on oral health, and the preferred frequency of treatment provision between groups of patients receiving scale and polish at 6-, 12-, and 24-month intervals. The null hypothesis was that there would be no difference in the responses between trial groups.

## Methods

### Design

The trial protocol was reviewed and approved by Cheshire Local Research Ethics Committee (reference Q/1506/100.) Trial registration was with UKCRN (ID 5101); and ISRCTN (ISRCTN56889016). Core funding of the trial was provided by The Oral Health Unit of the National Primary Care Research and Development Centre at The University of Manchester. Cheshire and Merseyside Comprehensive Research Network (funded by the National Institute of Health Research) awarded research support costs.

The study was a randomised 3-armed parallel trial with an allocation ratio of 1. Recruited dental practice patients were recalled for routine dental check-ups and provision of scale and polish (according to group allocation) over a period of 2 years; this 24-month follow-up equates to the maximum period advised between routine dental recall appointments [10].

### Participants

Participants were recruited from three large family dental practices in North West England. Patients, aged 18–60 years, scheduled for a routine dental check-up were sent an information leaflet about the trial and allocated an appointment during a specified trial recruitment session. Individuals who were interested in trial participation were screened by independent trial examiners to ensure that they fulfilled the eligibility criteria (Table 1). Participants were healthy, regular dental attenders with no systemic risk factors for periodontal disease [11] and no clinical evidence of significant periodontal disease. The latter was characterised by the participants’ Basic Periodontal Examination (BPE) sextant codes being less than 3 [12]. Written consent was secured from all participants and they were free to withdraw this, without giving reason for their decision, at any time during the follow-up period.

**Table 1:** Participant inclusion and exclusion criteria

• **Inclusion Criteria**	• Male or Female regular/routine attenders at dental practice
	• History of previous examinations and scale and polish
	• Aged 18 – 60 years
	• Good general health
	• 20+ permanent teeth (including crowned teeth)
• **Exclusion Criteria**	• BPE code of 3 or more in one or more sextants and/or requirement for more extensive periodontal therapy
	• More than 3 actively carious teeth
	• Requirement for prophylactic (pre-scaling) antibiotic cover
	• Removable prosthesis or orthodontic appliance present
	• Existing systemic condition which poses a risk factor for periodontal health e.g. diabetes mellitus
	• Medication which is known to affect the appearance or health of the periodontal tissues
	• Immunosuppressant state
	• Pregnancy
	• Involvement in any concurrent trial

The trial was powered to detect a difference in the primary outcome measure of gingival bleeding. A pragmatic approach to sample size calculation was taken as there were no available data to estimate an effect size. Specialist advice was taken and a suite of power calculations, based upon expected prevalence of gingival bleeding at follow-up (the primary outcome measure), was carried out. In order to detect clinically significant differences in proportions of participants with gingival bleeding, and allowing for 20% loss to follow-up, 369 patients were required to consent to participate in the trial.

Participants were stratified according to the presence or absence of supragingival calculus deposits at baseline and the trial manager used minimisation [13,14] to generate the random allocation sequence. Participants’ allocated interventions were revealed to them, when they returned for their first 6-month appointment after randomisation, by the hygienist providing the trial intervention. The independent examiners who collected data were employees of the salaried dental service with no connections to the practices and were blind to the allocation. The participants were asked not to reveal or discuss their allocation with the examiners or their family dentist. The statistician who carried out data analysis was also blind to group allocation.

### Interventions

All participants received a single-visit scale and polish at baseline. This was carried out by one of nine practice hygienists and therapists, all of whom were appropriately qualified, registered with the regulatory body in the UK (General Dental Council) and experienced in delivery of scale and polish. The definition of single-visit scale and polish used by Beirne *et al.*[6] was used to ensure a standardised approach to treatment delivery. This involved full-mouth sub- and supra-gingival scaling, carried out with an ultrasonic scaler, to remove accretions and stain from teeth. In cases where participants were unable to tolerate ultrasonic instrumentation, hand scaling instruments were used. After scaling, teeth were polished using an air motor-powered rotary rubber cup and polishing paste. Root planing was not undertaken and mouth rinses (or any other chemotherapeutic agents) were not used [6].

**Figure 1:**
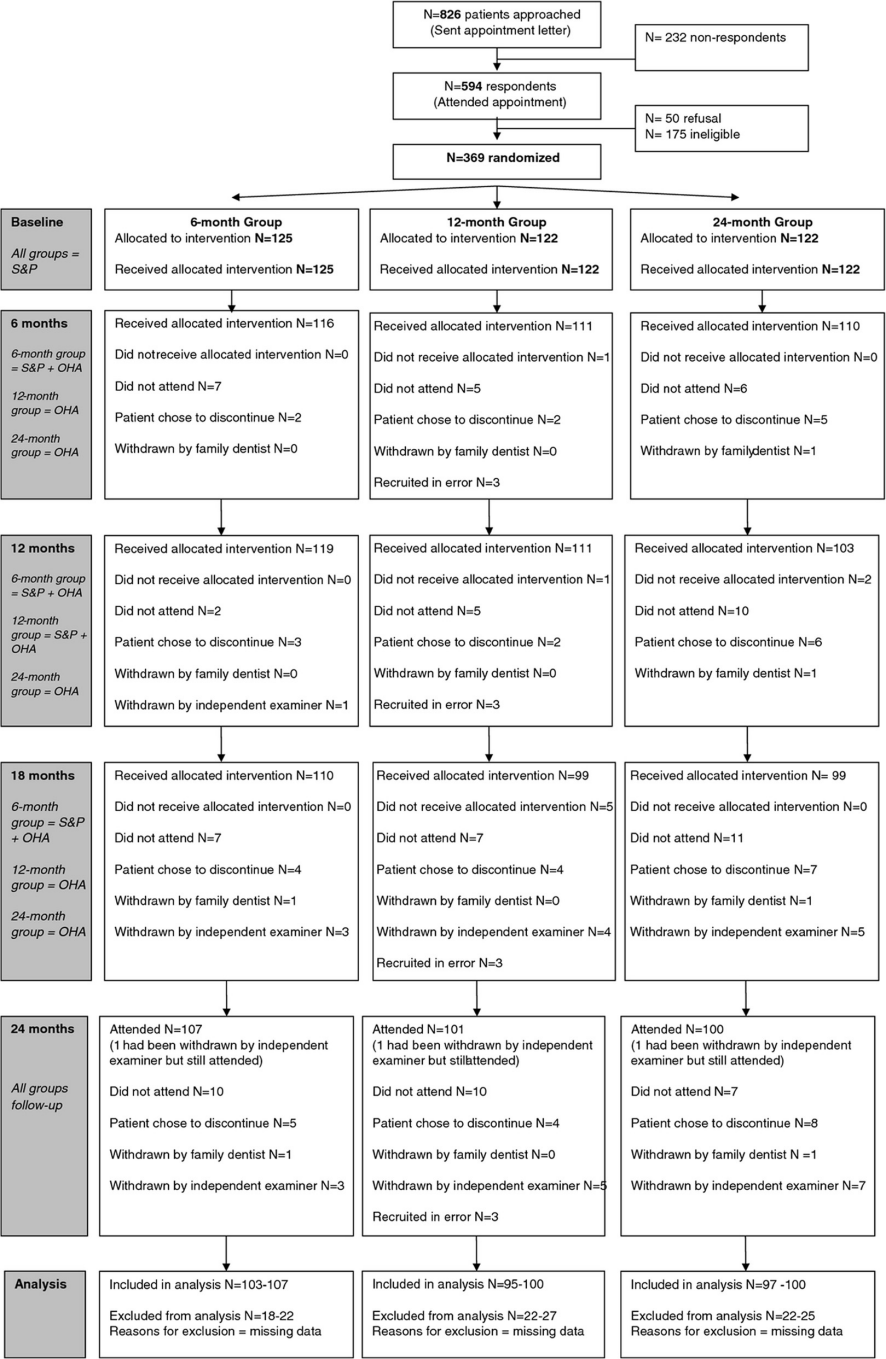
Consort RCT flow diagram: patient reported outcomes.

Participants were recalled for dental check-ups with their family dentist at 6-monthly intervals, which included monitoring their periodontal health using BPE. Immediately after their dental appointment they attended a hygienist or therapist for standardised oral hygiene instruction [15] and their trial intervention. One group represented the traditional routine 6-monthly regime (6-month group) and received single-visit scale and polish at each recall appointment (6-, 12-, and 18-months.) A second group (12-month group) received scale and polish at the 12-month recall appointment only. The third group (24-month group) did not receive a scale and polish after baseline (Figure 1).

### Outcomes

Measurements were taken at baseline and 24-month follow-up. Independent trial examiners asked patients to assess their oral cleanliness on a five-point scale in response to the question, *“How clean does your mouth feel on a scale of 1 to 5, where 1 is the least clean you could imagine and 5 is the cleanest you could imagine?”* Data were recorded on specially designed paper forms in the surgery.

A short, self-completed questionnaire was delivered in the practice waiting area prior to patients undergoing their baseline and follow-up examinations. Participants were asked to rate the importance of scale and polish (using a 5-point scale) for oral cleanliness; maintenance of gum health; prevention of halitosis; prevention of dental decay; and “whiteness” of teeth. Scores ranged from '1′ indicating 'of no importance at all’ to '5’ indicating 'extremely important’.

### Analysis

The groups were labelled to conceal the identity of the intervention and permit blind statistical analysis. PASW Statistics 18 [16] and STATA 11.0 for Windows [17] were used for data analysis.

A Kruskal-Wallis test was used to compare ordinal outcomes between groups at follow-up. This data included subjectively-reported levels of oral cleanliness, and questionnaire responses. The subjectively-reported oral cleanliness scores were dichotomised: participants scoring 4, or 5 were defined as reporting a 'High’ level of oral cleanliness. Similarly, the questionnaire data regarding frequency of scale and polish was dichotomised: 'Every six months or more frequently’; and 'Every year or less frequently’. A chi-squared test was used to compare the binary outcomes between treatment groups at follow-up. Logistic regression, adjusted for the baseline values of each outcome, was used to calculate the odds ratios for binary outcomes. An ordered logit model (procedure *ologit*) [17] was used to estimate the relationship between the ordinal outcomes and the treatment group, adjusted for corresponding baseline values.

Multiple imputation (n = 100 imputations) [18] was performed using *mi logit* procedure for binary outcomes and *mi ologit* procedure for ordinal outcomes [17] with the following variables used in imputation: corresponding baseline values; participant gender; participant age at baseline; deprivation index; and randomisation group.

Both complete dataset and multiple imputation analyses are presented in Tables 2, 3, 4, 5 and 6. The 6-month group is the reference group given that it represents the 'traditional’ frequency at which scale and polish is provided (often in conjunction with routine dental examination.)

**Table 2:** Baseline characteristics of trial participants

**Characteristic**	**6-month group**	**12-month group**	**24-month group**
**Baseline allocation**	125	122	122
**Age (years )**			
Mean (SD)	37.1 (10.4)	39.6 (10.8)	36.4 (10.6)
**Gender**			
N (%) Male	57 (45.6)	43 (35.2)	34 (27.9)
**IMD Quintile**^**a**^			
N (%)			
1 Most Deprived	40 (32.0)	40 (32.8)	34 (27.9)
2	29 (23.2)	29 (23.8)	30 (24.6)
3	18 (14.4)	18 (14.8)	24 (19.7)
4	24 (19.2)	21 (17.2)	21 (17.2)
5 Least Deprived	14 (11.2)	14 (11.5)	13 (10.7)
**Smoking history**^**b**^			
N (%)			
Never	83 (66.4)	70 (57.4)	71 (58.2)
Past	21 (16.8)	31 (25.4)	29 (23.8)
Current	12 (9.6)	15 (12.3)	15 (12.3)
Missing	8	6	7
**No. of Teeth present**			
Mean (SD)	27.8 (2.4)	27.8 (2.1)	27.6 (2.3)
Missing	0	1	0
**Decayed Teeth**			
N (%) any	9 (37.5)	8 (33.3)	7 (29.2)
Missing	0	1	0
**Filled Teeth**			
Mean (SD)	7.7 (4.5)	7.7 (4.7)	6.8 (4.3)
Missing	0	1	0

^a^ IMD derived from participants’ postcodes. Quintiles based on national standards.

^b^ Self reported smoking status. For participants recruited 02/2006 – 09/2006 these data were reported retrospectively, at the 12-month recall. For all other patients, smoking data were reported at baseline.

**Table 3:** Baseline patient-reported data (participants who provided information at both baseline and follow-up)

**Patient-reported outcome**		**6-month group**	**12-month group**	**24-month group**
Level of oral cleanliness (N = 307)				
1 (Least clean)		1 (0.9)	0	0
2		6 (5.6)	10 (10.0)	7 (7.0)
3		45 (42.1)	51 (51.0)	51 (51.0)
4		46 (43.0)	34 (34.0)	37 (37.0)
5 (Cleanest)		9 (8.4)	5 (5.0)	5 (5.0)
'High’^c^ level of oral cleanliness at baseline (N = 307)				
N (%) scoring 'High’		55 (51.4)	39 (39.0)	42 (42.0)
How important do you think a regular scale and polish is to keep your mouth clean? (N = 298)	1 (No Importance)	3 (2.9)	10 (10.4)	8 (8.1)
	2	17 (16.5)	12 (12.5)	8 (8.1)
	3	25 (24.3)	17 (17.7)	24 (24.2)
	4	19 (18.5)	22 (22.9)	23 (23.2)
N (%)	5 (Extremely important)	39 (37.9)	35 (36.5)	36 (36.4)
How important do you think a regular scale and polish is to keep your gums healthy? (N = 302)	1 (No Importance)	3 (2.9)	5 (5.1)	2 (2.0)
	2	5 (4.8)	2 (2.0)	6 (6.1)
	3	11 (10.5)	4 (4.1)	10 (10.1)
	4	24 (22.9)	24 (24.5)	23 (23.2)
N (%)	5 (Extremely important)	62 (59.0)	63 (64.5)	58 (58.6)
How important do you think a regular scale and polish is to prevent bad breath?	1 (No Importance)	11 (10.5)	13 (13.5)	9 (9.1)
	2	17 (16.2)	9 (9.4)	13 (13.1)
	3	24 (22.9)	24 (25.0)	22 (22.2)
	4	16 (15.2)	20 (20.8)	26 (26.3)
(N = 300)	5 (Extremely important)	37 (35.2)	30 (31.3)	29 (29.3)
N (%)				
How important do you think a regular scale and polish is to make your teeth whiter? (N = 297)	1 (No Importance)	14 (13.5)	16 (16.8)	8 (8.7)
	2	18 (17.3)	17 (17.9)	11 (11.2)
	3	22 (21.2)	16 (16.8)	26 (26.5)
	4	24 (23.1)	23 (24.2)	30 (30.6)
N (%)	5 (Extremely important)	26 (25.0)	23 (24.2)	23 (23.5)
How important do you think a regular scale and polish is to prevent tooth decay? (N = 299)	1 (No Importance)	3 (2.9)	2 (2.0)	3 (3.1)
	2	7 (6.8)	11(11.2)	3 (3.1)
	3	6 (5.8)	10 (10.2)	15 (15.3)
	4	23 (22.3)	9 (9.2)	22 (22.5)
	5 (Extremely important)	64 (62.1)	66 (67.4)	55 (56.1)
N (%)				
How often do you think you need a scale and polish? (N = 300)	Once every 3 months	19 (18.3)	19 (19.2)	21 (21.7)
	Once every 6 months	49 (47.1)	48 (48.5)	47 (48.5)
N (%)	Once a year	22 (21.2)	29 (29.3)	24 (24.7)
	Once every 18 months	2 (1.9)	1 (1.0)	0 (0.0)
	Once every 2 years	8 (7.5)	1 (1.0)	5 (5.2)
	Less than every 2 years	4 (3.7)	1 (1.0)	0 (0.0)

^c^'High’ level of oral cleanliness is defined as a participant reporting a score of 4 or 5.

**Table 4:** Subjectively assessed oral cleanliness at follow up

	**6-month group**	**12-month group**	**24-month group**	**Statistical test**
Level of oral cleanliness^d^ (N = 307)				Kruskal-
1 (Least clean)	0	2 (2.0)	3 (3.0)	Wallis Test
2	9 (8.4)	3 (3.0)	12 (12.0)	
3	42 (39.2)	48 (48.0)	55 (55.0)	P = 0.004
4	51 (47.7)	43 (43.0)	23 (25.0)	
5 (Cleanest)	5 (4.7)	4 (4.0)	5 (5.0)	
'High’^e^ level of oral cleanliness at follow-up (N = 307)				Χ^2^ test
N (%) scoring 'High’	56 (52.3)	47 (47.0)	30 (30.0)	P = 0.003
Complete dataset analysis (N = 307)				
Odds Ratio from Logistic regression	1.00	0.95	0.40	-
(95% CI) for follow up adjusted for baseline high level of oral cleanliness		(0.53, 1.70)	(0.22, 0.74)	
Multiple imputation (ITT) analysis (N = 368)				
Odds Ratio from Logistic regression	1.00	0.94	0.39	-
(95% CI) for follow up adjusted for baseline high level of oral cleanliness		(0.53, 1.66)	(0.21, 0.73)	

^d^ Assessed by asking participant, *“How clean does your mouth feel on a scale of 1 to 5, where 1 is the least clean you could imagine and 5 is the cleanest you could imagine?”*.

^e^'High’ level of oral cleanliness is defined as a participant reporting a score of 4 or 5.

**Table 5:** Patient questionnaire data: importance of scale and polish at follow-up

		**Patient-reported score**	**6-month group**	**12-month group**	**24-month group**	**Kruskal-Wallis test**
						**P-value**
**How important do you think a regular scale and polish is to keep your mouth clean?**	Follow-up	1 (No Importance)	10 (9.7)	5 (5.2)	5 (5.1)	0.502
	(N = 298)	2	10 (9.7)	13 (13.5)	7 (7.1)	
		3	27 (26.2)	21 (21.9)	30 (30.3)	
	N (%)	4	12 (11.7)	12 (12.5)	20 (20.2)	
		5 (Extremely important)	44 (42.7)	45 (46.9)	37 (37.4)	
	Complete dataset analysis (N = 298)	OR (95% CI)^f^	1.00	1.28	0.97	-
				(0.74, 2.19)	(0.58, 1.63)	
	Multiple imputation (N = 363)	OR (95% CI)^f^	1.00	1.29	0.96	-
				(0.75, 2.20)	(0.57, 1.60)	
	Follow-up (N = 302) N (%)	1 (No Importance)	3 (2.9)	2 (2.0)	0 (0)	0.234
		2	2 (1.9)	2 (2.0)	3 (3.0)	
		3	9 (8.6)	7 (7.1)	17 (17.2)	
**How important do you think a regular scale and polish is to keep your gums healthy?**	N (%)	4	20 (19.0)	14 (14.3)	16 (16.2)	
		5 (Extremely important)	71 (67.6)	73 (74.5)	63 (63.6)	
	Complete dataset analysis	OR (95% CI)^f^	1.00	1.25	0.79	-
	Complete dataset analysis (N = 302)	OR (95% CI)^f^				
				(0.65, 2.40)	(0.43, 1.45)	
	Multiple imputation	OR (95% CI)^f^	1.00	1.28	0.81	-
				(0.68, 2.42)	(0.44, 1.47)	
	(N = 367)					
**How important do you think a regular scale and polish is to prevent bad breath?**	Follow-up	1 (No Importance)	14 (13.3)	7 (7.3)	8 (8.1)	0.477
	(N = 300)	2	12 (11.4)	10 (10.4)	10 (10.1)	
		3	27 (25.7)	21 (21.9)	27 (27.3)	
	N (%)	4	10 (9.5)	17 (17.7)	15 (15.2)	
		5 (Extremely important)	42 (40.0)	41 (42.7)	39 (39.4)	
	Complete dataset analysis (N = 300)	OR (95% CI)^f^	1.00			
				1.64	1.20	-
				(0.97, 2.78)	(0.71, 2.01)	
	Multiple imputation	OR (95% CI)^f^	1.00	1.67	1.20	-
				(0.99, 2.83)	(0.99, 2.83)	
	(N = 367)					
**How important do you think a regular scale and polish is to make your teeth whiter?**	Follow-up	1 (No Importance)	18 (17.3)	15 (15.8)	10 (10.2)	0.490
	(N = 297)	2	15 (14.4)	12 (12.6)	8 (8.2)	
		3	24 (23.1)	25 (26.3)	35 (35.7)	
	N (%)	4	23 (22.1)	20 (21.1)	19 (19.4)	
		5 (Extremely important)	24 (23.1)	23 (24.2)	26 (26.5)	
	Complete dataset analysis (N = 297)	OR (95% CI)^f^	1.00	1.13	1.25	-
				(0.68, 1.87)	(0.76, 2.07)	
	Multiple imputation (N = 366)	OR (95% CI)^f^	1.00	1.14	1.26	-
				(0.70, 1.86)	(0.76, 2.09)	
**How important do you think a regular scale and polish is to prevent tooth decay**	Follow-up (N = 299)	1 (No Importance)	4 (3.9)	1 (1.0)	1 (1.0)	0.298
		2	5 (4.9)	4 (4.1)	5 (5.1)	
		3	8 (7.8)	9 (9.2)	12 (12.2)	
	N (%)	4	18 (17.5)	13 (13.3)	20 (20.4)	
		5 (Extremely important)	68 (66.0)	71 (72.4)	60 (61.2)	
	Complete dataset analysis (N = 299)	OR (95% CI)^f^	1.00	1.71	1.03	-
				(0.89, 3.26)	(0.57, 1.86)	
	Multiple imputation (N = 364)	OR (95% CI)^f^	1.00	1.79	1.07	-
				(0.94, 3.44)	(0.60, 1.90)	

^f^Proportional Odds Ratio from Ordered Logistic regression for follow up adjusted for baseline values of each outcome.

**Table 6:** Patient perceptions of scale and polish frequency at follow up

	**6-month group**	**12-month group**	**24-month group**	**Statistical analysis**
**How often do you think you need a scale and polish?**				
**Follow-up (N = 300)**				
**N (%)**				
Once every 3 months	19 (18.3)	16 (16.2)	15 (15.5)	Kruskal-Wallis
Once every 6 months	62 (59.6)	55 (55.6)	48 (49.5)	P = 0.194
Once a year	20 (19.2)	21 (21.2)	26 (26.8)	
Once every 18 months	0	3 (3.0)	4 (4.1)	
Once every 2 years	1 (1.0)	1 (1.0)	4 (4.1)	
Less frequently than every 2 years	2 (1.9)	3 (3.0)	0	
**Follow-up dichotomised data (N = 300)**				
**N (%)**				
Every 6 months or more				Χ^2^ test
frequently^g^	81 (77.9)	71 (71.7)	63 (64.9)	P = 0.126
Every year or less frequently^h^	23 (22.1)	28 (28.3)	34 (35.2)	
**Complete dataset analysis (N = 300)**				
Odds Ratio from Logistic	1.00	1.70	2.89	-
regression (95% CI) for follow-up adjusted for baseline frequency		(0.80, 3.59)	(1.36, 6.13)	
**Multiple imputation (N = 366)**				
Odds Ratio from Logistic regression	1.00	1.76	3.09	-
(95% CI) for follow-up adjusted for baseline high level of oral cleanliness		(0.80, 3.86)	(1.33, 7.20)	

^g^Defined as a participant reporting a required scale and polish frequency of 'once every 3 months’ or 'once every 6 months’;

^h^Defined as a participant reporting a required scale and polish frequency of 'once every year’, 'once every 18 months’, 'once every 2 years’ or 'less frequently than every 2 years.’

## Results

A CONSORT diagrammatical representation of participant flow [19] is presented in Figure 1. Participants were recruited over 40 dedicated sessions scheduled at the participating family dental practices during the periods February 2006 – June 2006; and June 2007 – September 2007. Of the 826 patients approached, 369, (44.7%) gave consent and were randomly allocated to an intervention group. The baseline characteristics of trial participants, and baseline patient-reported data, are presented in Tables 2 and 3 respectively. In total, 308 (83.5%) of those recruited attended both baseline and follow-up appointments; 281 participants (76.2%) attended all five trial appointments. Seventeen participants (4.6%) were withdrawn from the trial so that additional periodontal treatment could be carried out by their family dentist. Another 17 (4.6%) chose to discontinue their participation in the trial.

At follow-up (Table 4), participants in the 24-month group were significantly less likely (P = 0.003) to report a 'high’ level of oral cleanliness (30%, OR 0.40; 95% CI 0.22, 0.74) compared to the 6-month group (52.3%). There was no significant difference between the 12-month group (47%, OR 0.95; 95% CI 0.53, 1.70) and the 6-month group. The multiple imputation analysis gave similar results.

The distributions of patient-reported scores for importance of scale and polish with respect to oral health (clean mouth, healthy gums, fresh breath, white teeth, prevention of caries) were not significantly different between groups at follow-up (Table 5.)

The majority of participants at follow-up (77.9%, 71.7% and 64.9% in the 6-month, 12-month and 24-month groups respectively) reported that they required a scale and polish every 6 months or more frequently (Table 6.) The difference between these proportions was not statistically significantly different (Chi-squared P = 0.126). The odds ratios for the binary outcomes (adjusted for baseline values) suggest that being in the 24-month trial group increased the likelihood of choosing a scale and polish interval of every year or less frequently (OR 2.89; 95% CI 1.36, 6.13).

## Discussion

This study is the first published practice-based randomised controlled clinical trial investigating single-visit scale and polish provision with long-term follow-up [8]. It is the first practice-based trial to investigate views of patients receiving scale and polish at different frequencies. The measures and questionnaires used for data collection have not been validated; however, the information provided by participants increases our understanding of patient views and preferences regarding scale and polish provision. The findings of this study suggest that a large majority of dental attenders, who attend routinely and who have no significant periodontal disease, believe that professionally-delivered scale and polish is important for maintenance of their oral health, dental appearance, and social acceptability (in terms of fresh breath). Participants who did not receive a single-visit scale and polish during the follow-up period (i.e. 24-month group) were significantly less likely to report 'high’ levels of oral cleanliness than those participants who received the treatment more frequently.

The patient-reported oral cleanliness scores indicate that participants positively associate 6-monthly, professional scale and polish intervention with a 'high’ level of oral cleanliness. The clinical outcomes of this trial, reported elsewhere do not support the subjectively-reported oral cleanliness results [8]. The clinical outcome measurements did not detect statistically or clinically significant differences in the prevalence of the primary outcome, gingival bleeding, nor the secondary outcomes (plaque and calculus) between trial groups. With respect to hard and soft deposits at follow-up, prevalence of plaque was greater than 70%, and prevalence of calculus was greater than 54% in all trial groups with non-significant differences between groups [8]. There is, therefore, a disparity in the patients’ perceptions of the benefits of scale and polish, and the normative clinical findings.

The results of the patient questionnaire support previous findings which indicate that patients believe that scale and polish will keep their gums healthy, stop tooth decay, make their mouths feel good and improve their appearance [9]. The majority of participants surveyed thought that scale and polish was important to prevent oral health from deteriorating and for their mouths to be aesthetically and socially acceptable. The objective clinical findings of the RCT are not definitive given that this was a preliminary practice-based trial and had associated limitations; nevertheless, the null findings raise questions about the benefits of 6-monthly scale and polish over less frequent treatment provision and suggest a requirement for larger trials to enable more comprehensive clinical examinations and longer follow-up periods [8]. However, the subjective (patient-rated) results obtained suggest that even if scale and polish was shown to have little clinical benefit; or 12- or 24-month provision was shown to have the same benefit as the more traditional 6-monthly scale and polish, patient demand for routine scale and polish at 6-monthly intervals would persist.

### Limitations of the practice-based trial

Barriers to carrying out high quality research in a practice-based setting have been identified, including reductions in routine clinical activity and significant opportunity costs for practices hosting research studies [20,21]. The issues which may have impacted upon the design, conduct and findings of the clinical trial are discussed in detail elsewhere [8]. This paper focuses solely on secondary, patient-reported, outcomes not accounted for in the sample size calculation.

With respect to the patient-reported outcomes, criticism could be levied against the measures used, as these were simple, non-validated 5-point scales. However, there were no validated subjective measures in existence at the time the trial was designed and the content of the study questionnaire was based on findings of other trials in the literature [9].

### Scale and polish associated with six-monthly dental recall

The evidence base for 6-month frequency of dental check-ups in adult patients is tenuous [22,23] and whilst guidelines [10] have been introduced to encourage dental practitioners to extend the recall intervals for patients with low risk of dental disease, the 6-month dental check-up remains the 'norm’ for most routine dental practice attenders. The result is that half of all courses of dental treatment in England consist of examination with or without diagnostic radiographs and scale and polish services [1,24,25]. The results of the questionnaire indicate that healthy adult patients are keen to attend their dental practitioner twice-yearly and that they may be accustomed to routinely receiving a scale and polish in association with their routine check-up; most participants at follow-up reported a perceived need for scale and polish 'Every 6 months’ (54.6% of total participants. 59.6% of 6-month group; 55.6% of 12-month group; and 49.5% of 24-month group). One might assume it is likely that throughout their lifetime, patients have become conditioned that 6-monthly dental attendance is 'best’; this social construct may have been affected by factors such as parental attendance, dentists’ advice (this itself possibly influenced by historical patterns of remuneration) [26], advertising campaigns [23] and societal customs or norms [22].

Currently, there is no convincing evidence to support (or refute) the scale and polish intervention or the frequency of its provision (often 6-monthly) to maintain or improve periodontal health [6]. If future studies can find no difference in risk or benefit when scale and polish is provided at intervals longer than 6 months, this questions the legitimacy of routinely recalling healthy adults twice-yearly for a dental check-up and scale and polish and reinforces the need for increased recall intervals for low-risk patients [10]. Whilst the 6-monthly scale and polish has become embedded in dentists’ and patients’ consciousness, routine dental recall and associated provision of scale and polish for healthy individuals has opportunity costs: dentists could use the time spent providing scale and polish in other ways. In the current political and economic climate, state-funded healthcare systems, including the NHS, have a responsibility to ensure resources are deployed efficiently to maximise health benefits, reduce health inequalities, and increase productivity and access to services [27,28]. From a professional and ethical position, privately-paying patients and those co-paying for publically-funded dental services should be presented with all the available evidence to enable them to make informed choices about their treatment options.

In this trial, participants in the 24-month group were more likely to feel they needed scale and polish every year or less frequently. It is possible that receiving a less frequent scale and polish regime 'enforced’ as a result of trial participation influenced participants in the 24-month group to feel that they needed this treatment less frequently. However, it must be remembered these patients were recalled at 6-monthly intervals during the trial for a dental check-up with their 'family dentist’ (and therefore incurred similar opportunity costs to the 6-and 12-month scale and polish groups.) In England, NHS non-exempt dental patients currently pay the same treatment charge for their dental check-up whether or not they have a scale and polish; so under the current contract, there appears to be little financial benefit to the NHS if intervals between scale and polish for low risk patients are extended but the dental recall period remains 6-monthly. Had dental recall frequency been adjusted in accordance with trial scale and polish provision, patient-reported findings may have been different. However, modifying recall intervals (and remuneration for treatment provision), and economic analysis of treatment frequency was not a feature of this preliminary trial; further research including investigation of these issues is required before confident conclusions can be drawn.

#### Implications for evidence-based practice

Evidence-based practice requires clinicians to make decisions which are underpinned by a combination of available evidence, their own experience and patients’ wishes [29]. Given a lack of evidence to support (or refute) effectiveness and optimal frequency of treatment [6], the more subjective components of the model (practitioner experience, and patient preference) are naturally given more weighting and are more likely to influence the clinical decision-making process with respect to provision of scale and polish. However, even if multiple high quality trials fail to demonstrate a benefit for 6-monthly scale and polish over less frequent provision, it is likely that practitioners and patients will still hold the same strong beliefs and preferences, based on experience and traditional practice. Therefore, if definitive research evidence regarding clinical effectiveness were to bring about amendments to NHS policy regarding scale and polish provision, such change may be viewed with scepticism [26]. Guidelines and policy changes which challenge long-held clinical beliefs may arouse suspicion amongst clinicians that these are politically-driven, cost-cutting exercises and a threat to professional autonomy [30]. The way in which individuals view a particular situation will influence the weightings of the components of the evidence-based care model; entrenched views of dentists regarding benefits of scale and polish and strong patient demand for the treatment may supersede definitive evidence leading to tension between policy-makers and dental professionals. This being the case, policy makers would be wise to consider views of all stakeholders including dental professionals and patients alongside definitive evidence as part of any decision-making processes regarding future provision of scale and polish.

## Conclusions

This trial raises important issues regarding patient perceptions of scale and polish and the required frequency of its provision.

Scale and polish appears held in high regard by regularly-attending healthy adult patients; and receiving the treatment at different frequencies during the trial did not change this. However, groups who had longer intervals between scale and polish imposed upon them appeared to perceive that the treatment could reasonably be provided annually or less frequently.

Currently, in the absence of a strong evidence base to support (or refute) the clinical effectiveness of single-visit scale and polish, the beliefs and preferences of patients regarding scale and polish are likely to be influential drivers for treatment provision.

A requirement for further research investigating different aspects of scale and polish provision has been previously highlighted. In addition, the effects of various methods of oral hygiene instruction on oral health, as adjuncts to the professional clinical intervention, require further investigation [31].

As the evidence base for scale and polish develops to a stage at which clear guidelines can be developed, it is important that policy-makers engage appropriately with dental professionals and patients as part of the decision-making process. A combination of appropriate communication, support, and professional incentives will be required to overcome barriers and facilitate any future proposed changes to primary care based (state-funded) scale and polish provision.

## Competing interests

The authors declare that they have no competing interests.

## Authors’ contributions

Martin Tickle and Keith Milson were responsible for the initial research question. Clare Jones, Martin Tickle, Keith Milson, and Tatiana Macfarlane drafted the study protocol which detailed the trial methodology. The delivery of the trial within the practices, and the collection and management of trial data was the responsibility of Clare Jones. Philip Ratcliffe and Annette Wyllie both contributed clinical and practical information for the development of the trial protocol; and both assisted in the operational management of the study. Tatiana Macfarlane, the trial statistician, was responsible for the design and conduct of the analyses. This paper was initially drafted by Clare Jones and Martin Tickle following which all the authors provided comments. All have read and approved the final version.

## Pre-publication history

The pre-publication history for this paper can be accessed here:

http://www.biomedcentral.com/1472-6831/13/50/prepub
